# Measuring Electrical Responses during Acute Exposure of Roots and Rhizoids of Plants to Compounds Using a Flow-Through System

**DOI:** 10.3390/mps5040062

**Published:** 2022-07-18

**Authors:** Robin Lewis Cooper, Matthew A. Thomas, Rachael M. Vascassenno, Kaitlyn E. Brock, David Nicholas McLetchie

**Affiliations:** 1 Department of Biology, University of Kentucky, Lexington, KY 40506-0225, USA; matt137thomas@gmail.com (M.A.T.); rachael_vascassen@mymail.eku.edu (R.M.V.); kaitlynbrock@uky.edu (K.E.B.); mclet@uky.edu (D.N.M.); 2 Department of Biology, Eastern Kentucky University, Richmond, KY 40475-3102, USA

**Keywords:** roots, electrophysiology, flow-though

## Abstract

Monitoring electrical signals in plants allows the examination of their acute and chronic physiological changes and responses to stimuli. Understanding how plant roots/rhizoids respond to chemical cues in their environment will provide insight into how these structures acquire resources. Chronic exposure to L-glutamate alters root growth and is known to alter Ca^2+^ flux inside roots. The ionic flux can be detected by electrical changes. A rapid and relatively easy approach is presented to screen the electrical sensitivity of roots/rhizoids to compounds such as amino acids and known agonists/antagonists to receptors and ion channels. The approach uses a background-flow system of basal salt or water; then, the administered compounds are added to the roots/rhizoids while monitoring their electrical responses. As a proof of concept, the response to flow-through of glutamate (1 mM) was targeted at the root/rhizoids of three plants (*Arabidopsis thaliana*, *Pisum sativum* and *Marchantia inflexa*). Both *Arabidopsis thaliana* and *Pisum sativum* produced rapid depolarization upon exposure to glutamate, while *M. inflexa* did not show an electrical response. In some experiments, simultaneous recordings with impedance measures for acute changes and glass electrodes for chronic electrical potential changes were used. The effect of potassium chloride (300 mM) as a depolarizing stimulus produced responses in both *P. sativum* and *M. inflexa.* The protocol presented can be used to screen various compounds in a relatively rapid manner for responsiveness by the roots/rhizoids of plants.

## 1. Introduction

Measuring electrical responses in plants is common practice. As with animals, the approaches used for measuring electrical signals are very diverse. Various approaches and potential applications of measuring electrical responses, which are applicable to many plants, have been reviewed [1,2,3]. In brief, plant electrical measurements include (1) extracellular signals around a plant (i.e., field potentials in the air or soil or in other mediums), (2) potential differences, in and out of a plant, to an environment (i.e., differences between a fluid compartment within a plant and external surfaces such as the soil or a moist surface), and (3) intracellular recordings across a cell wall to fluid compartments (i.e., xylem or phloem) or to the surface of a plant.

Depending on the research questions, and for ease of reproducibility, a standardized recording method is optimal. In this report, two general methods are presented to monitor the responses of intact seedling roots (or rhizoids of a liverwort) to acute exposure to chemical agents. There are various approaches used in placing a reference electrode (i.e., a ground for completing the electrical circuit) to measure potential differences and changes within a plant. However, depending on where the reference electrode is placed, the signals may be dampened in amplitude due to dissipation of the electrical field. The approach of placing a second glass electrode as a reference or ground lead into the plant may provide the largest response to detect a change in the electrical potential within a plant. Dipping a leaf into a beaker of KCl with a reference lead placed inside the beaker is another approach. This is an approach for larger plants where a beaker can be placed within the recording area; however, spatially, this is cumbersome for seedlings. Growing seedlings on a moist filter paper and placing a reference lead on the moist filter paper in contact with the roots is an alternative approach. Depending on the concentration of the salts bathing the filter paper, the amount of contact the roots have with the filter paper and the distance from the roots to the ground wire can result in varied amplitudes of electrical responses. The filter paper approach also assumes that an ionic change can be measured through the roots to the bathing salt solution on the filter paper. In addition, if various compounds are introduced on the roots, the ionic nature of the compound can produce an electrical potential change which can result in an electrical artifact; thus, this possible electrical change must be accounted for. One challenge of working with small plants is ensuring the circuit is complete. We found that placing a ground wire on the base of the root from the sprout, with a small amount of conductive paint containing a silver paste, provides a good electrical connection with a plant. A solution can be perfused over the roots without a change in the environment in direct contact with the reference lead which serves as a ground, thus illustrating how a plant responds to alterations in the environment, selectively on the roots. There are various approaches used for detecting the electrical signals of plants, depending on the plant and responses being examined [4].

In nature, the chemical concentrations in the soil are dynamic and roots/rhizoids are exposed to many compounds in different concentrations and for different periods of time. How a plant responds to a particular substance can be determined by growing seedlings in a media and adding or removing a substance of interest. To address whether plants respond to repetitive exposure, as well as whether the responses show desensitization to a compound, an approach using repetitive acute exposure needs to be considered. The amino acid L-glutamate is known to alter root growth and causes Ca^2+^ flux inside roots, and such fluxes are detected by electrical changes [5,6]. Using L-glutamate, two different flow-through approaches are presented as a proof of concept for detecting the acute responses of the roots of seedlings or rhizoids of a liverwort. 

The purpose of this report is to illustrate the flow-through approaches we found useful for monitoring acute chemical exposure to roots/rhizoids by detecting the electrical signals within plants.

## 2. Procedure

### 2.1. Plant Material 

*Arabidopsis thaliana* plants were obtained from the Arabidopsis Biological Resource Center (https://abrc.osu.edu/researchers cat# CS22574 9 October 2021) and followed their germination protocol. Plants were grown in silica sand saturated with half-strength basal salts. Five-week-old plants were used. *Pisum sativum* (pea) plants (Sugar Daddy from BURPEE^®^; Home Depot, Lexington, KY, USA) were planted in moist soil. Seven days after germination, plants were removed from the soil and placed on moist (with half-strength basal salts commonly used for *A. thaliana*) paper towels in a 100 × 15 mm Petri dish. Plants were used within 24 h of being placed on the paper towels. *Marchantia inflexa* Nees & Mont were field-collected from multiple sites on the island of Trinidad, Republic of Trinidad and Tobago in 2018 and cultured in an environmentally controlled chamber (Percival, T-35LL). Plant fragments (thalli 5 to 7 mm long and ca. 4 mm wide) were placed in 35 × 10 mm Petri dishes on Whatman^®^ # 1 filter paper. Plants were watered with distilled water or with half-strength basal salt. Half-strength basal salt, as suggested for *A. thaliana*, was used for culturing and for examining electrical responses. Water for dissolving the basal salts was deionized. 

All plants were grown in the laboratory @ 22 C and under white LED lights (12 h dark/12 h light of ca. 40 µmol m^−2^ s^−1^). 

### 2.2. Electrical Recordings

Measurements of the electrical response within the stems of *P. sativum*, the leaf of *A. thaliana*, or the thallus of *M. inflexa* were performed by inserting a glass microelectrode (catalogue # 30-31-0 from FHC, Brunswick, ME, 04011, USA, with the tip broken to a jagged opening in the range of 10 to 20 µM diameter) into the respective plant structure. The electrode was filled with 0.3 mM KCl. A ground wire, used as a reference lead, was placed on the base of the stem next to the attached cotyledon for the *P. sativum*, on a leaf of *A. thaliana*, and on another part the thallus (at least 4 mm away for the glass microelectrode) of *M. inflexa* with a small drop of conductive paint (Bare conductive ^®^, online bareconductive.com, 14 June 2022). The surfaces of the plants were dried where the silver paint was placed. The silver wires of the recording (i.e., inside the glass electrode) and ground leads were coated with chloride by using concentrated bleach for about 20 min to obtain the Ag-Cl coating. All wires were rinsed thoroughly with water prior to being used. The glass electrode was placed within the stem, leaf, or thallus with a micromanipulator under a dissecting microscope. The electrode was inserted 1 to 2 mm into the stem of the *P. sativum*, just across the first cell layer of the leaf in *A. thaliana*, and in the thallus of *M. inflexa*. The recordings were performed within a grounded Faraday cage. To reduce the 60 Hz noise from the pump, a common ground wire with an Ag-Cl coating was placed into the beaker with the basal salt that was used for the background perfusion.

The electrical signals were obtained using an amplifier (Neuroprobe amplifier, A-M systems; obtained from ADInstruments, Colorado Springs, CO. 80906 USA) and connected to a computer via an AD converter (4 s Power lab 4/26, ADInstruments, Colorado Springs, CO. 80906 USA). Recordings were performed at an acquisition rate of 20 kHz. Events were observed and analyzed using software Lab-Chart 8.0 (ADInstruments, USA). 

### 2.3. Flow-Through Techniques

**Figure 1:**
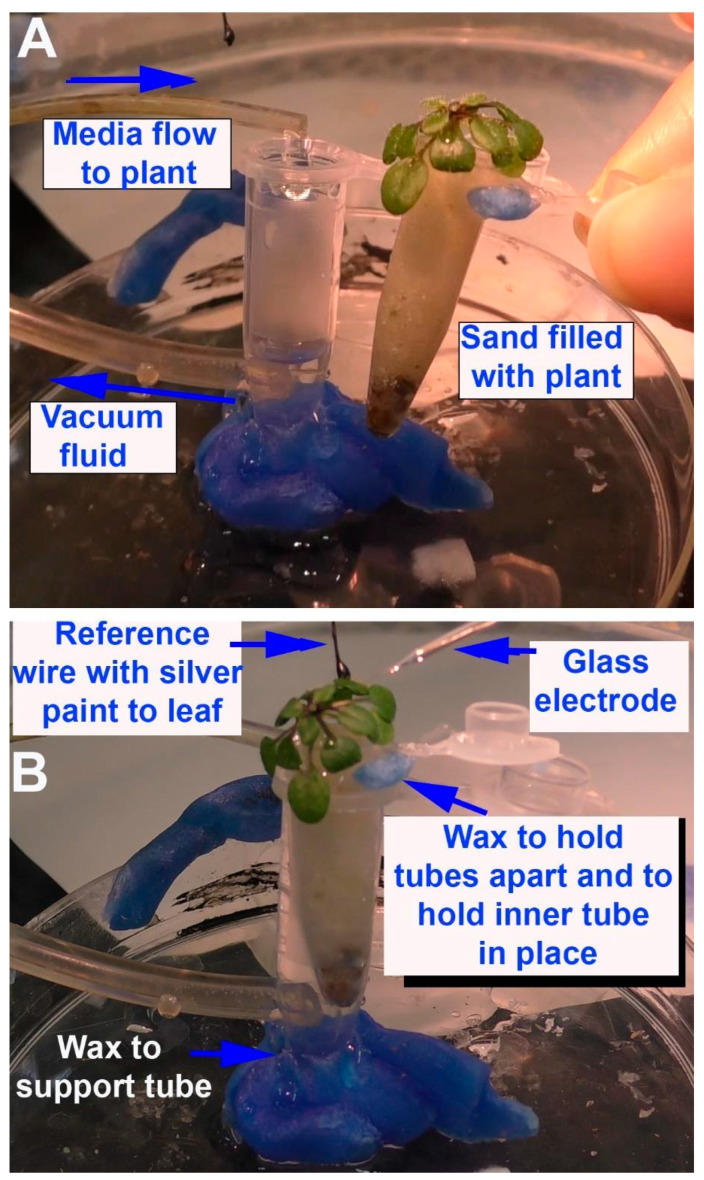
Illustration of the flow-through setup using *Arabidopsis thaliana*: (**A**) The outer 1.5 mL Eppendorf tube is waxed in place on a plastic Petri dish. The drip rate for the flow-through solution is tested over the 1.5 mL Eppendorf tube and the suction at the bottom is provided to be sufficient in not allowing the fluid inside to rise; (**B**) the smaller 0.5 mL Eppendorf tube along with *A. thaliana* is placed inside the outer 1.5 mL Eppendorf tube and held in place with wax, allowing a small space for air to circulate around the tube. The reference wire is placed on a leaf and conductive paint ensures electrical contact. The base of the stem of one leaf is chosen for the glass recording electrode to be placed.

Two approaches are presented for the acute exposure of roots to compounds. One approach was used for very small seedlings and small roots of *A. thaliana*. These were grown on sand in a 0.5 mL Eppendorf tube with a hole in the bottom. Turface^®^ (Profile Products LLC Buffalo Grove, IL 60089, USA)) was added to the bottom of the tube to prevent the sand from falling out. This tube was then placed in a 1.5 mL Eppendorf tube. The larger 1.5 mL tube was then filled with basal salt that permeated up through the hole at the bottom of the smaller 0.5 mL tube. This design allowed for quick replacement of the basal salts. A small amount of surgical wax held the tubes together, so they would not move during a recording session. A hole was made on the side of the outer microcentrifuge tube close to the bottom, and the end of tubing was placed within the hole for a snug fit. Suction was maintained in this tube to pull the solutions out of the larger tube and to promote gravity flow-through of the roots within the sand of the smaller microcentrifuge tube. The hole on the large microcentrifuge tube was made using a hot soldering iron (Figure 1). A tube delivering basal salt or basal salt and glutamate was placed over the edge of the small Eppendorf tube. The tube had an internal diameter of 1.6 mm, and the flow rate was adjusted to 20 mL/min by regulating the pump speed (Masterflex pump (Cole-Parmer, Vernon Hills, IL 60061 USA; model # 7014). A video of the procedures used is provided (Video S1: https://www.youtube.com/watch?v=3151I9q0WUc, 1 June 2022).

**Figure 2:**
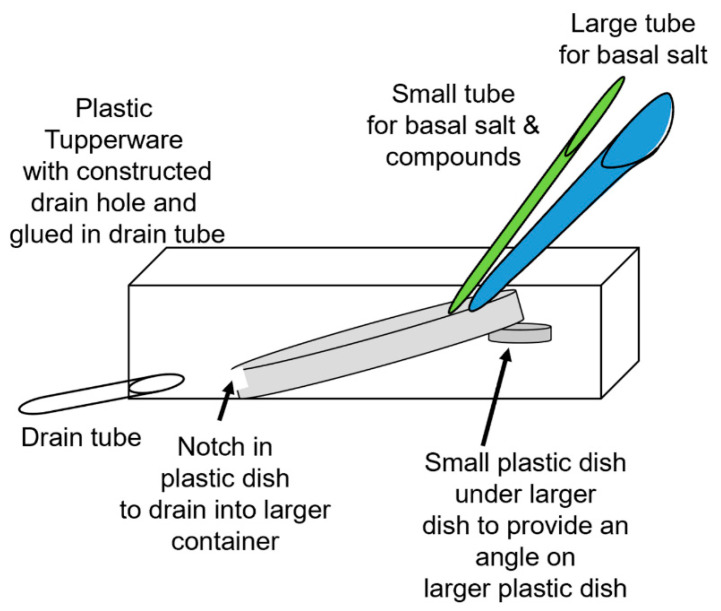
Schematic of the flow-through setup for *Pisum sativum* and *Marchantia inflexa*. The small green tube is for the basal salts and test compound and the large blue tube is to maintain a constant flow of basal salts. The large Petri dish (or a plastic dish) is tilted by setting it on a platform within a larger plastic Tupperware container. The notch in the edge of the larger Petri dish (about half the dish height) provides a dam for the solutions to bathe the roots and then drain into the larger container. A drain tube drains the Tupperware container. The Tupperware container can also be tilted by placing a wedge under one side to adjust the overall angle to control the rate at which the solution drains out of the Petri dish.

**Figure 3:**
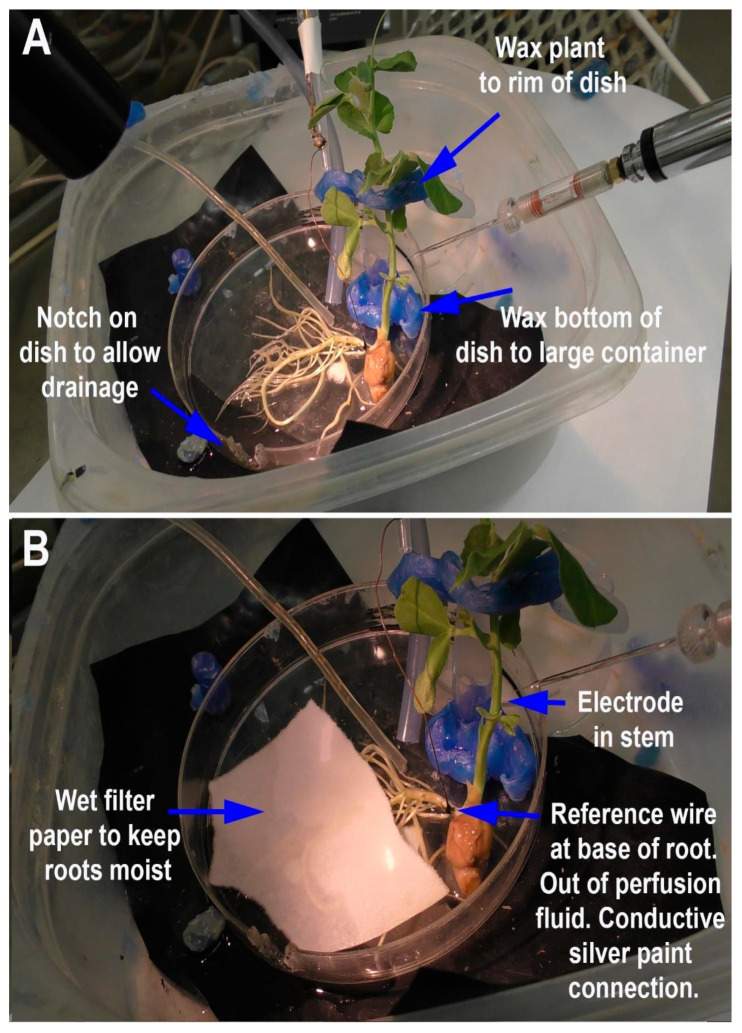
Illustration of the flow-through for *Pisum sativum*: (**A**) The plant is held in place by wax to prevent movement. Placement of the plant needs to be considered to provide ease of access to the stem for placing of the electrodes and of the tubes to provide solutions; (**B**) the smaller tube to provide the compounds to be tested is placed within the outflow of the background continuous basal salt flow-through from the larger tube. The reference wire is placed on the base and connected to the plant with conductive silver paint. The reference wire is to be placed in a way so as not to be in contact with the flow-through solutions. The recording electrode is placed in a stable location in the stem so vibrations of the plant will not disturb the recording. The filter paper is placed over many of the roots to maintain moisture levels, and to diffuse various solutions onto the roots.

Another approach was used for *P. sativum* with roots a few centimeters in length. The roots were removed from the soil in the starting tray and gently rinsed with half-strength basal salt. The roots were then placed in a 100 × 15 mm Petri dish with half-strength basal salt 24 h prior to recordings, with moist filter paper covering the roots to prevent the roots from drying out. The plants were then moved to the recording dish which was fixed in place. The recording dish was a 100 × 15 mm Petri dish maintained at a 30 to 40 degree slope and a notch was made at the lower side, allowing the compounds to drain. A larger plastic container on the floor collected the used solution (see schematic in Figure 2). The basal salt in which the roots were placed was used to provide constant flow-through via a constant stream above the roots at a rate of 80 mL/min, delivered by standard 14 L/S tubing (Cole-Parmer, Vernon Hills, IL 60061, USA). A smaller tube (ID 1.6 mm) was directed at a subset of roots with basal salt and switched to glutamate (1 mM) in basal salt at a rate of 40 mL/min (Figure 2). A setup using *P. sativum* in place with the reference and recording electrode, as well as the tubes for flow-through of the solutions, is shown in Figure 3A. The roots were covered with filter paper wetted with the same basal salt (Figure 3B). The solution for the smaller tube was switched back and forth from the glutamate solution to basal salt at various times to examine repetitive exposure to glutamate. The glutamate was flushed off the roots with the same basal salt as provided in the background flow-through. To examine if the disturbance from changing the tubing to the solution containing glutamate caused an electrical response, controls were performed by changing the solution to a control solution (basal salt). When changing solutions, the pump was not turned off and a small amount of air in the tube could be observed when the next solution was in contact with the roots. Thus, this allowed one to observe when glutamate was contacting the roots/rhizoids without the use of a dye. The responses of three model plants were examined for the proof of concept regarding the effect of glutamate on the electrophysical responses of the plant. The large tubing used a Masterflex Standard long-shaft pump (Cole-Parmer, Vernon Hills, IL 60061, USA) and the smaller tubing used a Masterflex pump (Cole-Parmer, Vernon Hills, IL 60061, USA; model # 7014.) (Video S2: https://www.youtube.com/watch?v=6J6G70iW2pM, 1 June 2022).

**Figure 4:**
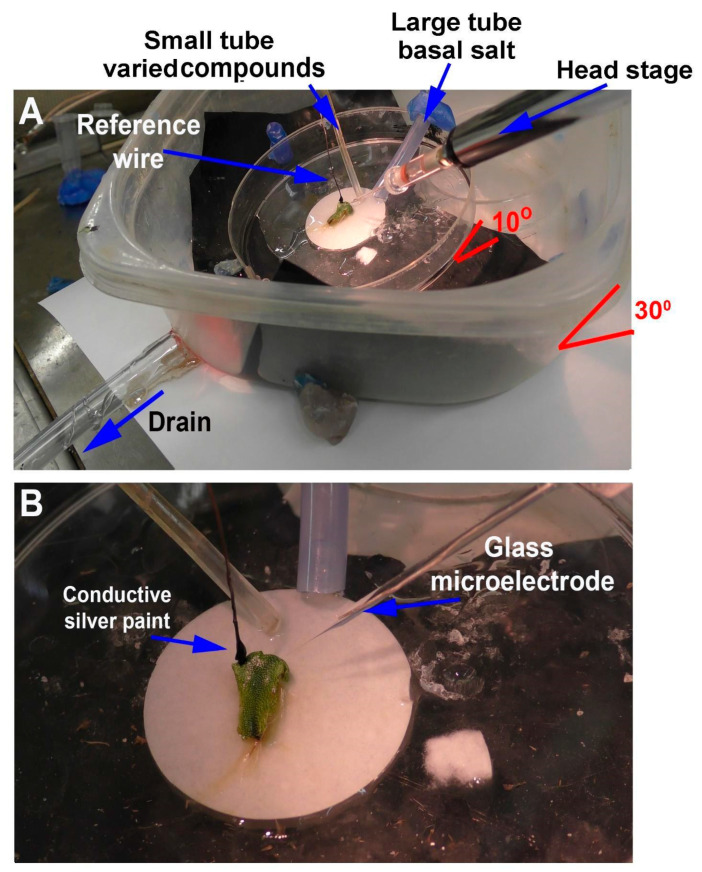
Illustration of the flow-through setup for *Marchantia inflexa*: (**A**) *M. inflexa* is low in the dish, so the large outer Tupperware container may need to be tilted slightly by placing a wedge under one side to allow ease in placement of the electrodes. The plant and the filter paper it was cultured on are placed in the middle of the Petri dish with the solutions to flow over the rhizoids placed on the paper, which helps to hold the filter paper in place. The filter paper can also be waxed to the dish to prevent movement. Placement of the plant needs to be considered to provide ease of access to the thallus for placing the electrodes and the tubes to provide solutions; (**B**) the reference wire is placed on the thallus with conductive paint while also avoiding contact with the solutions being administered. The recording electrode is placed in the thallus, which is well anchored to the filter paper by its rhizoids.

The approach for the smaller plant, *M. inflexa*, was to leave the plant and its rhizoids on the small filter paper to which they attached and to place the filter paper into the larger Petri dish (Figure 4). Then, the larger tube was placed for constant flow-through further up on the dish so the basal salt would drain over the filter paper. The smaller tube, used to deliver varying compounds, was placed lower within the stream of the basal salt being delivered from the larger tube (Figure 4). A video of the procedures used is provided (Video S3: https://www.youtube.com/watch?v=dK-xxF2iZj8, 1 June 2022). 

### 2.4. Comparing the Responses with the Two Recording Techniques Obtained Simultaneously

In order to examine reproducibility in the response to exogenous compounds to the roots/rhizoids, the seeds of *P. sativum* were allowed to sprout for 2 days so that small amounts of the roots contacted the filter paper and only short stems would form. This reduced the amounts of the roots and branches exposed to the fluid flowing over them. *M. inflexa* did not produce a large network of rhizoids, even over a week in culture, but variation in the extent of the rhizoids was still obvious. Thus, it is difficult to control for the variation in the surface area to the exposure of compounds using the flow-through system. The two-to-three-day-old sprouts of *P. sativum* and the one-week-old freshly cultured *M. inflexa* were used to examine the amount of variation in the responses between recording techniques, and between plants of the same species, to a given stimulus. The roots of *P. sativum* were subjected to the flow of water, and then, two drops of KCl (300 mM or 1 M) were placed at the mouth of the tube creating a flow of water over the roots. A second stimulus was set up in new plants which exposed the roots with a one-minute flow of glutamate (1 mM) and with a constant background flow of basal salt. The two stimuli used for *M. inflexa* consisted of water background perfusion, and then, exposure to 1 min of basal salt flow. The second stimulus was the same as for *P. sativum*, with water as a background perfusion while exposing the rhizoids to 2 drops of KCl (300 mM or 1 M) by introducing the KCl into the flow of the water before the collective flow contacted the plant. In addition, to compare between recording techniques, simultaneous recordings of the two leads used for impedance measures and the method with a ground lead on the surface of the plant and a glass electrode penetrating the plants were obtained. 

**Figure 5:**
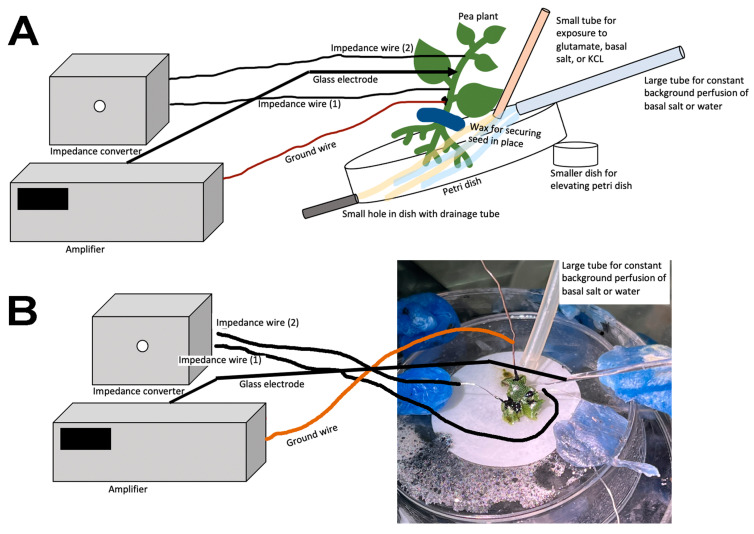
Illustration of the arrangement for simultaneous recordings of the impedance measure and the potential from an internal to an external measure with the glass electrode: (**A**) *Pisum sativum* and (**B**) *Marchantia inflexa*.

Representative illustrations of the recording arrangements are shown—in Figure 5A for *P. sativum* and Figure 5B for *M. inflexa*—for the simultaneous recording with the impedance measures and the glass electrode for the potential measures across the plant inner surface and outer surface. 

## 3. Results

The response to the flow-through of glutamate (1 mM) was used as a proof of concept to acutely expose the roots/rhizoids of the three plants, to examine whether the plants would show electrical response to exposure to glutamate. 

### 3.1. Arabidopsis thaliana

**Figure 6:**
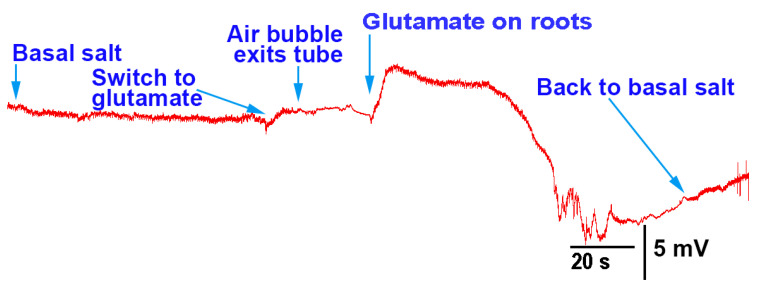
Electrophysiological recording from *Arabidopsis thaliana* during the flow-through of the roots with basal salt and glutamate. When glutamate (1 mM) came in contact with the roots, depolarization in the plant occurred.

The flow-through of the roots with glutamate for *A. thaliana* produced depolarizations (N = 6; *p* < 0.05 rank-sum sign test; Figure 6), and showed no response to basal salt being exchanged for basal salt as a control. The roots were in the sand within the tube which resulted in an even distribution over all the roots as compared to the roots of *P. sativum* in the Petri dishes, where the flow was directed over a subset of the roots. 

### 3.2. Pisum sativum

**Figure 7:**
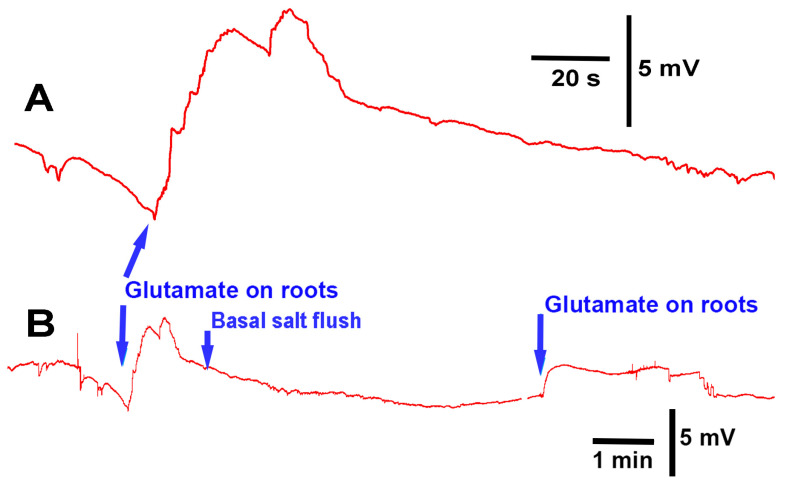
Electrophysiological recording from *Pisum sativum* during the flow-through of the roots with basal salt and glutamate: (**A**) when glutamate (1 mM) came in contact with the roots, depolarization in the plant occurred; (**B**) upon rinsing the glutamate away with basal salt, a second exposure to glutamate also produced depolarization. These traces are from the same recording, and different time scales are used to highlight the effects.

Upon exposure to glutamate (1 mM), *P. sativum* showed depolarization in its electrical response (N = 7; *p* < 0.05 rank-sum sign test; Figure 7A). After five minutes of flushing with basal salt, a second exposure to glutamate produced another instance of depolarization, indicating that the initial response did not produce long-term desensitization of the glutamate response (Figure 7B); moreover, it is likely that glutamate was able to flushed off the potential glutamate receptors.

### 3.3. Marchantia inflexa

**Figure 8:**
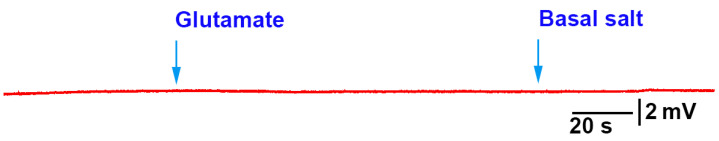
Electrophysiological recording from *Marchantia inflexa* during the flow-through of basal salt and glutamate on the roots. When glutamate (1 mM) came in contact with the rhizoids, no change in electrical response was measured.

In the protocol used, no electrophysiological response to glutamate was observed for *Marchantia inflexa* (N = 6, Figure 8). Future experiments examining the potential effects of various amino acids are planned for comparison among different plants, as well as assaying a range of concentrations. Perhaps different amino acids or varying concentrations of glutamate may reveal an electrophysiological response in *M. inflexa*.

### 3.4. Comparisons of the Impedance and Glass Electrode Measures

**Figure 9:**
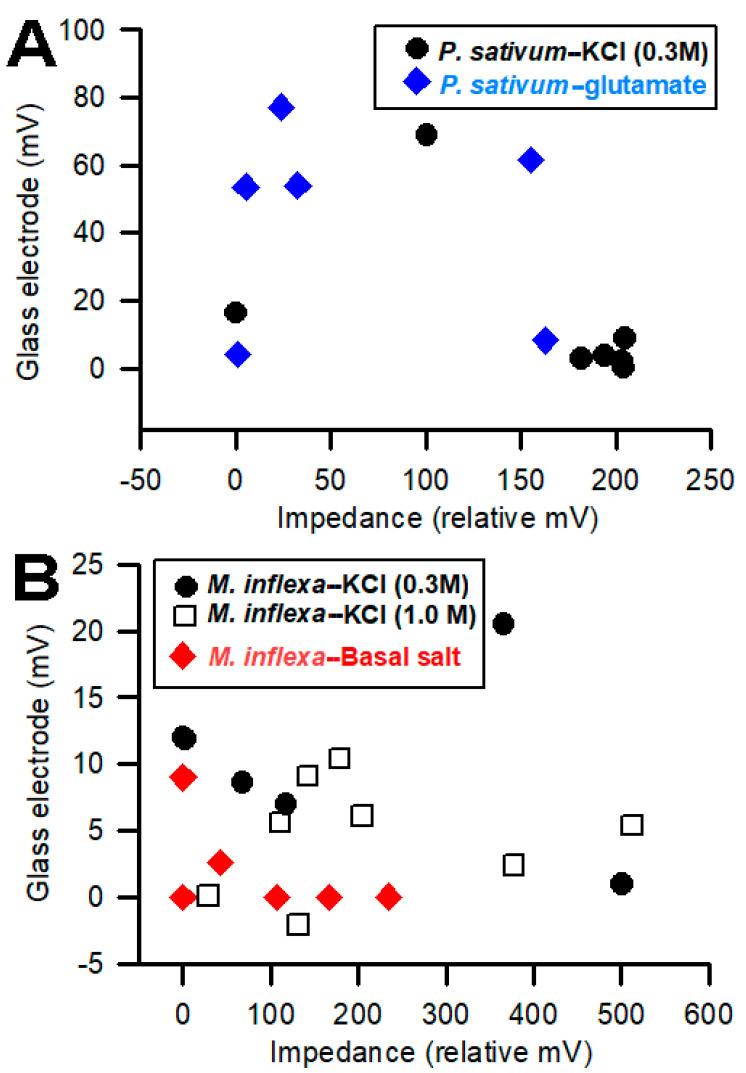
Comparisons of the amplitude of the responses obtained simultaneously from the plants using the two recording techniques: (**A**) Six *Pisum sativum* were examined for responses to KCl (0.3 M) and another six for responses to glutamate (1 mM). The impedance measures are relative as the amount of deflection in the traces was measured in voltage with a constant current source; (**B**) *Marchantia inflexa* was not as responsive to basal salt as KCl, so two different concentrations of KCl (0.3 and 1.0 M) were used for comparing the amplitude of the signals.

Comparing the electrical signals obtained with the impedance measures on the surface of a plant, compared to placing the impedance leads into the stem, showed that the amplitudes of the signals were larger when the leads were inserted into a plant. This was shown previously when recording in the stem of a tomato plant [4]. To assess whether the voltage measures made with the impedance converter correlated, in amplitude, with the glass electrode technique, the amplitudes were plotted for both *P. sativum* and *M. inflexa* with respect to the exposure to various stimuli. In some cases, for *M. inflexa*, brief exposure to KCl at 300 mM or to basal salt did not elicit a detectable change in electrical potential. Thus, the rationale was to examine whether a higher concentration of KCl (1 M) would provide large-enough signals to detect a change. Therefore, another set of *M. inflexa* was examined. For *P. sativum*, the response to basal salt was tested, and in a separate set of trials, the effect of exposure to 300 mM KCl was examined (Figure 9). In the plots showing the two recording techniques, there is a trend in the relationships (Figure 9).

## 4. Discussion

The methods presented can be used to investigate how roots/rhizoids may sense and respond to short- or long-term exposure to compounds in a controlled environment. As a proof of concept, three model plants were examined. In two of the three species, acute exposure to glutamate resulted in immediate electrical depolarization. The responses indicate the receptors to glutamate are present on the roots of *A. thaliana* and *P. sativum*, but not *M. inflexa*, under the experimental conditions used in this study. However, it was expected that the roots and rhizoids of plants would respond to a high extracellular concentration of KCl, similar to many animal cells. Cells are generally driven by potassium concentrations to maintain their resting membrane potentials; with exposure to extracellular KCl cells, they will typically depolarize cells, as was the case for *P. sativum* and *M. inflexa.* Simultaneous electrical potential recordings using the impedance technique and a glass microelectrode were obtained, illustrating that both techniques are possible to use for detecting responses to compounds. 

There was substantial variation in the electrical response among the same species of plant with regard to the same stimulus. This is likely due to the variation in root structure and contact area with the tested compounds. Different recording techniques may be required to monitor electrical signals, depending on the plant structure, such as stems with large leaves or sprouts. In addition, if the surface of the stem is easy to penetrate with an impedance lead or glass electrode, one might choose these approaches to maximize the detection of an electrical signal. A woody stem with high resistance to the internal fluid may not be well electrically connected to a lead placed on the surface, even with conductive paint; thus, penetration into a stem might be required. It is not expected that roots of the same species of plants would respond in the same manner as there is so much variation in root structure and fine processes of the root hairs. It may not be possible to expose the whole surface area equally and simultaneously; thus, it may be necessary to spread out the electrical signals, or even produce multiple deflections in the traces as the various roots come into contact with the solutions. However, a relative change in electrical potential can be measured due to introduced compounds. In addition, with the penetration of leads within a stem, it is difficult to know exactly which fluid compartment the leads are preferably measuring. Thus, variation in the responses between plants may occur due to comparing the responses in different compartments to a stimulus. Furthermore, penetrating a stem with a lead can induce an injury discharge to the plant, so some additional time may be required to obtain a stable baseline. It was shown in an aquatic plant (*Myriophyllum aquaticum*) that inserting an electrode generated an electrical response [7]. It is not surprising that an electrical response occurs when inserting an electrode into the stem of a plant. We also report that the insertion of the glass electrode induces an electrical change while first recording the electrical signals with leads for impedance and waiting for a stable response. However, in some cases, no differences in the impedance recordings were noted when the tip of the glass electrode penetrated a stem. In one recording, we did note a hyperpolarizing electrical signal from the stable baseline when the brief exposure to KCl (1 M) occurred for *M. inflexa*. There are various possibilities for this spurious response, such as the compartment within the stem in which the tip of the glass electrode might have been placed. Since the glass electrode is filled with an electrically conductive solution, it is possible that a slow change in the electrical potential occurs as some of the fluid in the glass electrode may leak into a plant. Small-tip glass electrodes can reduce leakage, but there is a trade-off as the small-tip electrodes tend to clog. A physiological response of the plant may even add to the blockage of the tip, depending on the cellular reaction to the electrode. It is common to use a 10 to 20 µm tip diameter and 0.1 to 0.3 M KCl for the electrical conductor for recording using glass electrodes in plants. However, one may wish to try different recording arrangements to determine what is optimal for the experimental design and questions being addressed [7]. It is known that an injury or physical disturbance to a plant, via a cut to a leaf or even the bending of a leaf, generates electrical signals which can be monitored using an impedance measure as well as with the insertion of a glass electrode in reference to the outside of the plant [8].

The development of root structures in *A. thaliana* is known to be altered by exposure to glutamate, a known agonist to glutamate receptors [5,9]. Developmental studies of roots with a compound embedded in a growth media, such as agar, presents constant exposure to the compound. Constant exposure could possibly result in a desensitization of the receptors. The family of glutamate receptors is well characterized physiologically, pharmacologically and by molecular make-up in animals. The metabotropic and ionotropic glutamate receptors in animals are generally classified by action of excitatory amino acids and by more selective agonists (i.e., kainic, N-methyl-D-aspartic (NMDA), and quisqualic acids, as well as selective antagonists) [10,11,12].

L-glutamate decreases the long growth of roots in *A. thaliana* and promotes branching, but the effects are not blocked by antagonist DNQX, MK-801 or AP-5, which are known to block ionotropic glutamate receptors in animals [6,13]. The exact classification of the glutamate receptor subtypes in the roots of various plants remains to be determined; however, for the plants examined, they appear similar to ionotropic glutamate receptors in animals [9,14]. Glutamate exposure produces a Ca^2+^ flux in plant roots though a glutamate-like receptor, as noted in some types of glutamate receptors in animals [15,16,17]. The view is that this response may aid the plant in detecting a nitrogen source. The rhizoids of *M. inflexa* did not appear express glutamate-like receptors in the culture conditions used in this study. Thus, glutamate responses do not appear to be universal in land plants. However, glutamate-like receptors might be expressed in other structures of *M. inflexa*. The genomic and proteomic analysis of different tissues of *M. inflexa* would help reveal the possibility of expression and location in a subtype of glutamate receptors.

The flow-through protocol presented allows acute and repetitive exposure to compounds. The ease of electrical recording with a reference electrode and conductive paint in contact with the plant, and a glass microelectrode placed within the plant, provides a means to measure ionic flow within the plant. In addition, cocktails of compounds can also be examined, as can agonists and antagonists, to examine potential types of receptors present on roots. The intracellular types of amplifiers for use in monitoring DC potentials over long periods are generally in the cost range of USD 5000, while measuring rapid changes or DC changes with the impedance converter, as used in this study, is in the range of USD 500–800. Both amplifiers require an analog-to-digital converter to be able to record to a computer. The glass electrode approach requires a manipulator and holder to aid in the placement of the glass electrode (~800 USD); thus, the impedance measures are cheaper to conduct with regard to equipment requirements.

It was anticipated that larger impedance measures would be correlated with larger signals obtained with glass electrodes. However, the opposite correlation occurred in *P. sativum*. In general, as the magnitude of the response increases for the impedance method, the magnitude of the glass electrode response decreases (Figure 9). For *M. inflexa*, there was no noticeable correlation in magnitude between the two techniques. This is surprising considering how close the two recording leads were to one another within the plant.

When observing the plot associated with *P. sativum* (Figure 9), the inverse correlation between the amplitudes/magnitudes for the two recording techniques allows us to pose interesting questions. If using two recording techniques simultaneously, will one of the recording methods present a more favorable path of conductivity—whether due to its proximity to the response site, or because of the corresponding compartment within which the recording electrode is placed—and, therefore, conduct a higher percentage of the plant’s total electrical response than the other recording electrode? Looking at the 0.3 M KCl flow-through for *P. sativum* in Figure 9, the four largest impedance measures for “*P. sativum*-KCl”, are associated with the four smallest glass electrode measures for the same compound. Correspondingly, when looking at glutamate flow-through for *P. sativum*, three of the four largest glass electrode measures produced three of the four smallest impedance measures for the same compound.

The exposure of roots/rhizoids to various compounds known in nature can occur for short periods of time with deluges and other natural phenomena, or over long periods of time as compounds containing organic materials decompose in the environment around a plant. These same methods could be used to study the long-term exposure of plants grown in soil, where soils might favor the retention of certain compounds over others. In addition, the examination of compounds exposed to roots while leaves may be photosynthesizing in different wavelengths, and/or being exposed to volatile substances, could be readily examined using the protocols and methods presented.

## Data Availability

Not applicable. The data are presented in the manuscript.

## Supplementary Materials

Video S1: Procedure to record from *Arabidopsis thaliana*. https://www.youtube.com/watch?v=3151I9q0WUc, 1 June 2022; Video S2: Procedure to record from *Pisum sativum.* https://www.youtube.com/watch?v=6J6G70iW2pM, 1 June 2022; Video S3: Procedure to record from *Marchantia inflexa.* https://www.youtube.com/watch?v=dK-xxF2iZj8, 1 June 2022.
